# Reflections on a pandemic: Disruptions, distractions and challenges of a clinical social worker on the frontline in New York City

**DOI:** 10.1177/1473325020981076

**Published:** 2021-03

**Authors:** Stephanie S Felder

**Affiliations:** National Catholic School of Social Service, The Catholic University of America, Washington, USA

**Keywords:** Mental health, reflexivity, reflective practice, critical reflection, social work practice

## Abstract

For many of us, COVID-19 markedly changed our world and how we operate in it daily. While the behavioral health ramifications of this pandemic are not fully known, they have clearly had an impact. For weeks, we all watched in disbelief as COVID-19 ambushed China, Italy, and other countries. When President Trump implemented the March 16, 2020, live broadcast detailing plans of how our nation would address COVID-19, we knew that it was just a matter of time before we began to experience what we saw happening around the world. Quickly, the escalation of COVID-19 in the United States caused a major shift for social work education, practice, and research. Social workers are serving in critical roles during this pandemic and providing care for COVID-19 patients and their families. The purpose of this article is to provide reflection on the disruptions, distractions, and challenges of a social worker serving in a leadership role on the frontlines at the Javits Center in New York during the COVID-19 pandemic.

As stated by the National Association of Social Workers (1999), the primary mission of the social work profession is to enhance human well-being and help meet the basic human needs of all people; the profession pays particular attention to the needs and empowerment of people who are vulnerable, oppressed, and living in poverty. For me, the mission of the social work profession was always a mantle to carry. I saw it as personal guidance from our social work leaders. In 2020, COVID-19 brought a new reality that elevated the mission of social work—a new reality for everyone. Indeed, the escalation of COVID-19 in the United States caused a major shift for social work education, practice, and research. The purpose of this article is to provide reflection on the disruptions, distractions, and discoveries of my experience serving at the Javits Center in New York during the COVID-19 pandemic.

## Disruptions

For weeks, we all watched in disbelief as COVID-19 ambushed China, Italy, and other countries, and brought the many disruptions of life which ensued. When President Trump implemented the March 16, 2020, live broadcast detailing plans of how the nation would address COVID-19, we knew that it was just a matter of time before we began to experience what we saw happening around the world. Since 2012, I have been a member of the United States Public Health Service (USPHS) Commissioned Corps. The purpose of the USPHS is to protect, promote, and advance the health and safety of the nation (United States Public Health Service, 2020). It consists of an elite team of over 6,500 uniformed health care professionals serving within duty stations in over 20 federal departments and agencies. Thus, I reacted to the COVID-19 appearance in the country with both an eagerness to serve and feelings of anxiety.

As a member of the USPHS, I served in leadership roles in 2017 for Hurricane Harvey, in 2018 for Hurricane Florence, and in two 2019 humanitarian missions along the U.S. border of Mexico. However, faced with the reality of COVID-19, I asked myself, are you really ready for this mission? I knew this would be a unique challenge, but I had no idea to what extent it would eclipse my earlier deployments. On March 25, 2020, I was notified that I would indeed deploy, and not only would I deploy but I would be going to the epicenter of the pandemic—New York City (NYC) to assist with a Federal Medical Station (the Javits Center).

So, what did this mean? The moment I had been preparing my husband and family for was upon us. Although my husband and I had been discussing what a COVID-19 deployment meant and how it might look, I found I had left many things undone that should have been done prior to this deployment (i.e., preparing a will). Also, the deployment was not the scenario we had prepared for; we never considered that I would be deployed to the epicenter, the place that would at some point reach over 170,000 COVID-19 cases with more than 14,000 deaths (New York City Health, 2020). When I told my husband I was being deployed, his first questions were where and when. When I responded, “New York City,” I saw his look of solemnness, but he (who had been in the Army) nodded his head that he understood—after all, this is what I signed up for, right?

Walking into New York on March 27, 2020, felt surreal; my mind flashed to my recent visit to the city for a Broadway show during the 2020 Valentine’s Day weekend. Then the city was alive, vibrant, and one could barely walk the streets; juxtapose that to this new reality. To see the city vacant made for a grim reality. The small particles of trash flying down the street in the wind seem to make time slow down as I began to understand the gravity of the mission.

## Challenges

### The magnitude of the mission

Once leadership began giving us an overview, I became aware that I would serve as the Behavioral Health Lead on behalf of the USPHS. What I did not understand at first, was that I was to be the Behavioral Health Lead for the USPHS, Army, Navy, and National Guard. We all would serve as one team composed of members from multiple uniformed service branches to provide care for both patients and staff at the Javits Center. The duties of the position began with delineating the mission for the integrated service behavioral health team that eventually involved over 30 service members including psychiatrists, psychiatric nurses, psychologists, licensed clinical social workers, and behavioral health technicians—and a therapy dog. In the end, our mission would be to provide behavioral health support to over 2,500 staff and patients within the Javits Center and to support mortuary affairs and outlying hospitals. In essence, it was our mission to ensure that the nurses, providers, and staff who entered the building and worked at Javits Center felt supported physiologically and psychologically so they could provide care initially to non-COVID-19 patients but, within days, to COVID-19 patients when the center was converted to serve only COVID-19 positive patients. The Javits Center had an operational capacity of 3,000 beds. Given that my prior experience was limited to providing clinical services at a federal medical station of 75 beds and 60 staff, this effort was massive, and the weight of the mission felt substantial.

As the number of staff members grew each day, we divided the work and decided which standard operating procedures were essential as patients entered the building. We also instituted procedures for behavioral health consults, psychotropic consults, patient de-escalation, and measures regarding potential death of patients or staff. We prepared space for all the people we would serve. In the end we provided over 6,000 behavioral health encounters with staff and patients providing an array of services (i.e., service member contacts, psych first aid presentations, dissemination of behavioral health materials, leadership/supervisory consultations, and psycho-education for staff members). In the end I found myself in the middle of a deployment that was, by far, the largest and weightiest of my career.

### Self-care during deployment

Amidst the many distractions happening during the deployment, maintaining self-care and focus was a priority for me. Each day when I woke up, I combined meditation with spiritual reflection. The task was simple; I wanted to maintain my health so that I could provide the upmost care to the staff who cared for patients, meet the social work needs of Javits Center patients, and eventually return to my family. I started a deployment journal, but many days I felt too tired to write and so a short 5-minute reflection about the day sufficed. I posed questions to myself such as: What went well? What didn’t go well? What could I have done better? And lastly, what do I need to do tomorrow? I wanted to end each day on a clean slate, so I could be better for the next day.

In the end, what kept me grounded were the daily calls to my family and friends. The texts and emails that wished me well, the prayers for my strength and well-being, and the scriptures that family members sent to let me know I wasn’t alone. What worried me the most was that I was in New York and not with them when they needed me the most. Questions whirled through my mind. What would happen if a family member became ill with COVID-19? What would I do? How could I manage this deployment if my own family fell sick? Fortunately for me, I never had to face any of those questions.

### Resiliency

What I learned from the Javits Center and from New York during COVID-19, is that` “resilience is that quality that permits some people to be knocked down by life and come back stronger than ever” (Larson, 2017: 34). The patients, staff, and everyone in New York were knocked down by COVID-19, but they stood back up. We all stood back up, taller, stronger, and together. One team, one mission, one fight every day. At the end of the day, neither the branch nor the color of the uniform mattered. What mattered was getting to the fight each day and winning. Sometimes our achievements were small (i.e., bringing in snacks on night shift or a plant for the wellness room to bring a little life to the center); sometimes the accomplishments were huge (i.e., releasing recovering patients to family members).

### Implications for mental health professionals

Managing self-care was a challenge for many health care professionals prior to COVID-19 (Centers for Disease Control (CDC), 2020). The pandemic has placed us all in an uneasy predicament—managing the care of others while maintaining our own care and livelihoods during a time of unknowns. As mental health professionals, we have an enormous dilemma ahead of us. Simply stated, one cannot serve from an empty vessel; one cannot give what one does not have. Self-care during this tumultuous time is paramount. The reality is that we still have our everyday stressors (i.e., marital problems, relationship issues, financial strain, prior physical and mental health issues). These current internal and external stressors will not stop because COVID-19 has arrive on the scene. Therefore, you will be caring for professionals and, in some cases, patients who are dealing with the onslaught of COVID-19 and, at the same time, the issues that plagued them prior to COVID-19. Most importantly, as the mental health professional, you may personally be dealing with similar issues.

During our time at the Javits Center, we encouraged all staff members located at the Javits no matter their duties to engage in self-care by:

- Encouraging providers, nurses, and operation staff to seek help early. We took a preventive stance by building rapport with staff and informing them of our behavioral health services rather than waiting for them to develop issues
- Creating a safe space for staff members that included one-on-one meeting areas and a room for yoga, meditation, and spiritual reflection
- Providing behavioral health materials centered on emotional fitness, sleep hygiene, managing anxiety, and caregiver stress
- Providing information on SAMHSA’s free, confidential 24/7 National Helpline available by phone or text at 1-800-662-HELP (4357) (Substance Abuse Mental Health Services Administration, 2020).
- Sharing a behavioral health wellness tip during meetings with Javits Center staff members to ensure they were aware of the mental health services and to provide helpful reminders about the importance of taking care of their own mental health.

## Conclusion

COVID-19 has changed how we all operate daily. While the behavioral health ramifications of this experience are not fully known, the pandemic has clearly had an impact. We are now entering into a phase of new normal. This new normal will look different for each of us, and the challenge will be to understand how we move forward in this new environment. We have witnessed impact on suicidal behavior, the financial strain experienced by the unemployed, and the ups and downs of the stock market (American Psychological Association, 2020; Centers for Disease Control (CDC), 2020). As social workers, we are now faced with the largest human service crisis of our times: clients with pre-existing mental health issues exacerbated by the stress associated with COVID-19 and persons with no previous behavioral health issues suffering from symptoms due to social isolation, quarantine, and the drastic change in daily life. We will see dramatic increases in depression, anxiety, substance abuse, grief and PTSD-like symptoms. As social workers, we are essential workers on the frontlines helping to meet the needs of COVID-19 patients—standing in the gap for our clients. One thing that the rest of the world can learn from New York is that resiliency is the best defense for COVID-19. Self-care and taking care of each other is paramount at this time. We all need time to reflect on this experience and examine the impact on our daily lives.

## References

[bibr1-1473325020981076] American Psychological Association (2020) COVID-19 and suicide. Available at: www.apa.org/monitor/2020/06/covid-suicide (accessed 8 December 2020).

[bibr2-1473325020981076] Centers for Disease Control (CDC) (2020) Coping with stress. Available at: www.cdc.gov/coronavirus/2019-ncov/daily-life-coping/managing-stress-anxiety.html (accessed 8 December 2020).

[bibr3-1473325020981076] Larson J (2017) Organizational and Process Reengineering Approaches for Health Care Transformation. 1st ed. USA: Productivity Press.

[bibr500-1473325020981076] National Association of Social Workers. (1999). *Code of ethics of the National Association of Social Workers*. Washington, DC.: NASW Press.

[bibr4-1473325020981076] New York City Health (2020) COVID-19 data. Available at: www1.nyc.gov/site/doh/covid/covid-19-data.page (accessed 8 December 2020).

[bibr5-1473325020981076] Substance Abuse and Mental Health Services Administration (SAMHSA) (2020) Disaster distress helpline. Available at: www.samhsa.gov/find-help/disaster-distress-helpline (accessed 8 December 2020).

[bibr6-1473325020981076] United States Public Health Service (2020) About us. Available at: https://usphs.gov/aboutus/v (accessed 8 December 2020).

